# Risk Factors Influencing Cognitive Function in Elderly Patients With Late‐Life Depression: A Scoping Review

**DOI:** 10.1002/brb3.70265

**Published:** 2025-01-09

**Authors:** Ping Jiang, Yunfeng Gao, Lin Wang, Xiaojun Shao, Lei Zhang, Gang Zhu, Li Duan

**Affiliations:** ^1^ Department of Health Management Research Chengde Medical University Chengde China; ^2^ Department of Orthopedic Surgery the Affiliated Hospital of Chengde Medical University Chengde China; ^3^ Department of Psychiatry the First Affiliated Hospital of China Medical University Shenyang China

**Keywords:** assessment tools, cognitive dysfunction, influencing factors, late‐life depression, scoping review

## Abstract

**Background:**

In recent years, cognitive impairment has emerged as a pivotal symptom in elderly patients with depression, exerting a substantial impact on the course and prognosis of diseases. Moreover, it persists even following remission from depression during the rehabilitation period. However, there remains an incomplete understanding of the relevant influencing factors for cognitive impairment in elderly depressed patients, which seriously impedes the development of risk prediction models and the subsequent research on precision intervention programs.

**Objective:**

The purpose of this study is to examine the current state of negative influencing factors and assessment tools for cognitive impairment in patients with late‐life depression (LLD), thereby providing a theoretical framework for the construction of subsequent targeted intervention programs.

**Methods:**

The search strategy employed in this study followed an evidence‐based approach, utilizing a systematic scoping review to thoroughly explore six English and four Chinese databases up until November 2023. Two researchers independently conducted article screening and employed thematic analysis to categorize the results into themes.

**Results:**

Following two rounds of rigorous screening conducted by the evidence‐based research team, data were meticulously extracted and succinctly summarized from five distinct themes encompassing socio‐demographic, physiological, psychological, genetic, and other related factors. In addition, a comprehensive compilation of 19 diverse assessment tools was undertaken. Ultimately, a total of 22 articles met the eligibility criteria for inclusion in this study. These comprised five longitudinal studies, nine pathological controlled studies, five cross‐sectional studies, two cohort studies, and one randomized controlled study.

**Conclusion:**

Cognitive dysfunction is an important symptom of LLD, which seriously affects the survival of patients. At present, the research on its influencing factors mainly includes socio‐demographic, physiological, psychological, genetic, and other related factors. There have been existing cognitive function assessment tools specifically for those 18‐ to 65‐year‐old patients of major depressive disorder, but there is still a lack of reliability and validity tests in LLD.

## Graphical abstract

Cognitive dysfunction is a pivotal manifestation of late‐life depression (LLD), significantly impacting the patient's overall quality of life. Hence, in order to investigate the risk factors and assessment tools for cognitive dysfunction in LLD, this study screened 22 articles meeting the inclusion criteria from six English databases and four Chinese databases. Ultimately, five key themes encompassing socio‐demographic, physiological, psychological, genetic, and other associated factors were extracted. Furthermore, a comprehensive complication of 19 distinct assessment tools was conducted.

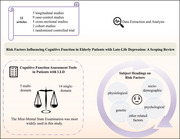

## 1 Introduction

Population aging emerges as a significant social challenge in the 21st century. It has been reported that the proportion of individuals aged 65 years and above is projected to reach 16% by 2025 (WHO 2019). Mental health plays a crucial role in the process of aging, with older adults exhibiting a heightened vulnerability to various mental disorders, including dementia (Reuben et al. 2024), depressive disorder (Steffens 2024), and anxiety disorders (Alwhaibi 2023). In particular, late‐life depression (LLD), second only to dementia, has become the most common mental disorder that seriously endangers the mental health of the elderly. It has a high risk of recurrence and seriously affects the quality of life of the elderly.

Substantial evidence suggests a high prevalence of cognitive impairment (ranging from 50% to 75%) in individuals with LLD, encompassing executive function, attention, and episodic memory deficits (Koenig et al. 2014). Furthermore, even during remission phases when depressive symptoms are alleviated, cognitive dysfunction often persists as a residual manifestation of depression. Notably, it was observed that despite achieving remission from depression, a staggering 94% of such patients remained impaired for at least 1 year (Bhalla et al. 2006). Consequently, cognitive impairment has gradually gained recognition as the fundamental symptom influencing the progression and prognosis of LLD (Rajtar‐Zembaty et al. 2022). The utilization of appropriate cognitive function assessment tools aid in identifying of cognitive impairment in LLD, while clarifying the risk factors associated with cognitive impairment which is crucial for delaying the recurrence of depression. Regrettably, despite some research advancements concerning cognitive impairment in patients with depression, a comprehensive review on relevant influencing factors and assessment tools specifically targeting the elderly population remains lacking.

The scoping review, which follows the principles of evidence‐based medicine, is a methodological framework for synthesizing knowledge and identifying evidence. Initially utilized in the field of medicine, it aims to consolidate the heterogeneity of existing research and facilitate literature preparation for subsequent scientific investigations (Tricco et al. 2018). In an endeavor to proactively address healthy aging and comprehensively manage disease‐related health risk factors in patients with LLD, this study systematically searched related databases to provide an overview of the influencing factors of cognitive impairment in this population as well as the current landscape of commonly employed cognitive function assessment tools. The objectives of this research are: (1) to optimize the selection of assessment tools for LLD and establish a theoretical foundation for designing specific assessment tools targeting cognitive function in LLD; (2) to provide a theoretical basis and clinical guidance for the development of intervention programs aimed at delaying the recurrence of depression.

## 2 Methods

### 2.1 Determining the Research Question

The establishment of an evidence‐based team was necessitated. All its members underwent rigorous training and achieved mastery in the research method of evidence‐based medicine, specifically scoping review. To be included, all studies were required to adhere to a predefined PCC model (Lockwood et al. 2019). P (participants), LLD patients; C (concept), cognitive function encompasses various conscious mental activities exhibited during wakefulness including memory, executive function, and other domains; C (context), encompassing hospitals, community settings, and other environments where LLD patients reside. Following an initial literature review and consultation with the research team, this scoping review was conducted to address the following research questions: (i) What is the current landscape of cognitive function assessment tools employed in patients with LLD? (ii) What are the contributing factors associated with cognitive impairment in LLD?

### 2.2 Search Strategy

The literature searches were conducted in APA PsycInfo, Web of Science, PubMed, Embase, Cumulative Index to Nursing and Allied Health Literature (CINAHL), Ovid, China National Knowledge Infrastructure (CNKI), China Science and Technology Journal Database (VIP), Wanfang Database, and SinoMed based on a combination of MeSH Terms and Free text words. Appendix A presents the retrieval strategy. All studies published up to November 2023 were included in this review.

### 2.3 Inclusion and Exclusion Criteria

Inclusion criteria are as follows: (i) the language was restricted to Chinese or English; (ii) participants were diagnosed with LLD; (iii) studies primarily focused on identifying factors influencing cognitive impairment in LLD patients; (iv) research methodologies predominantly utilized assessment tools such as scales and questionnaires for measuring cognitive function.

Exclusion criteria are as follows: (i) literature types included reviews, qualitative interviews, newspapers, editorials, conference abstracts, and other non‐research journal papers; (ii) literature that lacked access to original texts or complete data.

### 2.4 Study Selection

After importing the retrieval results into Endnote 20.0, two researchers systematically and gradually conducted literature screening by meticulously examining the title, abstract, and full text individually. In addition, they thoroughly scrutinized the reference lists of relevant articles. In instances of disagreement, a third member from the evidence‐based research team was consulted to engage in comprehensive discussion and ultimately determine whether to include or exclude the disputed studies.

### 2.5 Data Extraction and Analysis

The data was numerically categorized and summarized, and relevant information was extracted using Excel 2019 by two independent researchers. Ultimately, a comprehensive extraction of fundamental social demographic data (i.e., age, education, and sex), crucial analysis components (i.e., risk factors and the assessment tools), as well as other significant details were successfully accomplished.

## 3 Results

### 3.1 Data Reporting

**FIGURE 1:**
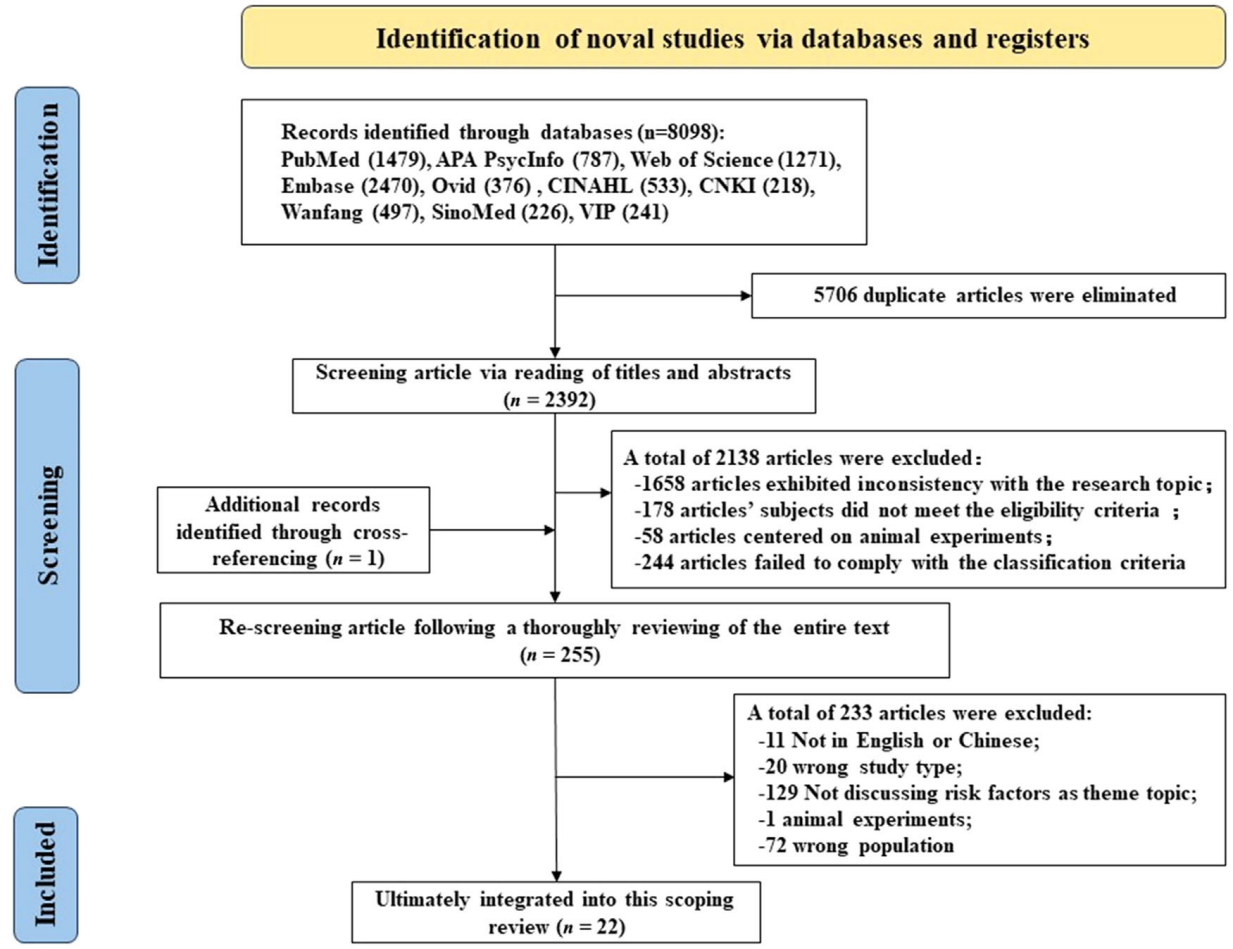
Flowchart of the paper selection process.

A total of 8098 articles were identified through database searching and additional articles were discovered using the snowball methodology by reviewing references (*n* = 1). Following two rounds of systematic screening, a final selection of 22 articles was made. Figure 1 presents an overview of the study selection process depicted in a PRISMA‐ScR flow diagram.

### 3.2 Fundamental Characteristics of Literature

This scoping review included a total of 18 articles in English and 4 articles in Chinese, encompassing patients from diverse settings including general hospitals, communities, and mental healthcare institutions. The sample sizes ranged from 62 to 2655 participants. The included research types comprised of five longitudinal studies (Gallagher et al. 2018; Manning, Chan, and Steffens 2017; Riddle et al. 2015; Steffens et al. 2020; Steffens et al. 2023), nine case–control studies (Huang et al. 2021; Morin et al. 2019; Wu et al. 2022; Zhang et al. 2021; Zhang et al. 2022; Zhong et al. 2018; Zhong et al. 2019; Zhou et al. 2021; Zhou et al. 2020), five cross‐sectional studies (Korten et al. 2014; Li et al. 2018; Shao et al. 2017; Yang et al. 2017; Yu 2013), two cohort studies (Funes et al. 2018; Naudé et al. 2014), and one randomized controlled trial (Rej et al. 2015). A comprehensive description of the included studies is provided in Table 1 in chronological order.

**TABLE 1:** Fundamental characteristics of the included literatures.

References	Location	Participants^a^	Study design	Cognitive function assessment tools	Influencing factors of cognitive impairment in LLD
Steffens et al. (2023)	USA	62	Longitudinal study	MMSE, TMT, SDMT, LM, CERAD	Neuroticism and stress
Zhang et al. (2022)	China	167/105	Case–control	MMSE, VFT, CDT, TMT, AVLT, DST	Odor identification
Wu et al. (2022)	China	123/70	Case–control	MoCA	Clinical symptoms of LLD
Zhang et al. (2021)	China	262/141	Case–control	MMSE, BNT, VFT, CDT, TMT, SDMT, AVLT, ROCF, SCWT	Neuropsychiatric symptoms
Zhou et al. (2021)	China	113/89	Case–control	MMSE, BNT, VFT, CDT, TMT, SDMT, AVLT, SCWT	Microvascular dysfunction
Huang et al. (2021)	China	116/100	Case–control	MMSE	Elevated homocysteine, brain‐derived neurotrophic factor, and neuregulin‐1
Zhou et al. (2020)	China	113/89	Case–control	MMSE, BNT, VFT, CDT, TMT, SDMT, AVLT, ROCF, SCWT	Elevated homocysteine
Zhong et al. (2019)	China	67/81	Case–control	MMSE	Cardiovascular diseases and related risk factors
Morin et al. (2019)	USA	95/41	Case–control	MMSE, BNT, VFT, WAIS, HVLT, TMT, SCWT	Financial capacity
Zhong et al. (2018)	China	67/81	Case–control	MMSE	Cortisol level, age
Li et al. (2018)	China	1276	Cross‐sectional	ROCF, MMSE	Female, age, rural or suburban residence, poor physical health status, education level of illiterate or elementary school, having no daily physical activity
Gallagher et al. (2018)	Canada	2655	Longitudinal study	MMSE, CDR	Age, sex, education, baseline cognition, hearing impairment, B_12_ deficiency, active depression within the last 2 years, and increased severity of depression
Funes et al. (2018)	USA	138	Cohort	MMSE, CVLT, TMT, ROCF, SCWT	Apathy, age, sex, education
Shao et al. (2017)	China	198	Cross‐sectional	MMSE	Age, living alone, smoking, prolonged/recurrent episodes, severity of baseline depression
Manning, Chan, and Steffens (2017)	USA	112	Longitudinal study	MMSE, TMT, SDMT, LM, CERAD	Vulnerability to stress
Riddle et al. (2015)	USA	299	Longitudinal study	MMSE, CERAD	Age, education, baseline depression severity, activities of daily living deficits
Rej et al. (2015)	Canada	130	RCT	MMSE	Age, lower interpersonal support, lower baseline global neuropsychological score, higher medical illness burden
Naudé et al. (2014)	Netherlands	369	Cohort	AVLT, DST, SCWT	Neutrophil gelatinase‐associated lipocalin
Steffens et al. (2020)	USA	271	Longitudinal study	CERAD	Age, education, genetic factors
Yang et al. (2017)	Taiwan, China	147	Cross‐sectional	MMSE	Methionine synthase 2756AA polymorphism
Korten et al. (2014)	Netherlands	378/132	Cross‐sectional	WAIS, AVLT, SCWT	Higher severity of psychopathology, first depressive episode, psychotropic medication, symptom dimensions
Yu (2013)	China	100/50	Cross‐sectional	VFT, CDT, TMT, SCWT, WCST, WMS	Education, sleep quality, and course of depression

Abbreviations: AVLT, Auditory Vocabulary Learning Test; BNT, Boston Naming Test; CDR, Clinical Dementia Rating Scale; CDT, Clock Drawing Test; CERAD, Consortium to Establish a Registry for Alzheimer's Disease; CVLT, California Verbal Learning Test; DST, Digit Span Test; HVLT, Hopkins Verbal Learning Test; LM, Logical Memory Test; MMSE, Mini‐Mental State Examination; MoCA, Montreal Cognitive Assessment; ROCF, Rey–Osterrieth Complex Figure Test; SCWT, Stroop Color and Word Test; SDMT, Symbol Digit Modalities Test; TMT, Trail Making Test; VFT, Verbal Fluency Test; WAIS, Wechsler Adult Intelligence Scale; WCST, Wisconsin Card Sorting Test; WMS, Wechsler Memory Scale.

^a^ Case–control group or ungrouped individuals.

### 3.3 Current Status of Cognitive Function Assessment Tools in Patients With LLD

A total of 19 different types of cognitive function assessment tools were utilized in the literature, as outlined in Table 2. The executive function exhibited superior performance with shorter completion time on the Trail Making Test (TMT) and Stroop Color and Word Test, while lower scores on alternative assessment tools indicated diminished cognitive function. Currently, one widely utilized and influential screening tool for cognitive function worldwide is the Mini‐Mental State Examination (MMSE) scale developed by Folstein, Folstein, and McHugh (1975), which aligns with the findings of this study.

**TABLE 2:** Current status of cognitive function assessment tools employed in the LLD population.

Assessment tool	Time (min)	Method of scoring	Property	Frequency (times)
MMSE	5–10	Higher scores indicate better cognitive function	Multi‐domain	17
BNT	< 5	Higher scores represent better language function	Single‐domain	4
VFT	< 5	Higher scores represent better language function	Single‐domain	6
CDT	< 5	Higher scores indicate better visuospatial function	Single‐domain	5
WAIS	60–120	Higher scores indicate better cognitive performance	Multi‐domain	2
HVLT	20–30	Higher scores represent better language function	Single‐domain	1
CVLT	/	Higher scores indicate better episodic memory	Single‐domain	1
TMT	< 5	A shorter completion time for the test indicates enhanced executive function	Single‐domain	9
SDMT	< 5	Higher scores indicate better attention abilities	Single‐domain	5
LM	/	Higher scores indicate better memory performance	Single‐domain	2
AVLT	/	Higher scores indicate better memory performance	Single‐domain	6
DST	< 5	Higher scores indicate better attention abilities	Single‐domain	2
CDR	45–60	The severity of cognitive impairment/dementia was rated by a healthcare professional	Multi‐domain	1
MoCA	Roughly 15	Higher scores indicate better cognitive function	Multi‐domain	1
ROCF	/	Higher scores indicate better visuospatial function	Single‐domain	4
SCWT	Roughly 5	Less time‐consuming indicates better executive function	Single‐domain	7
CERAD	60–120	Higher scores indicate better cognitive function	Multi‐domain	4
WCST	20–30	It is mainly used to evaluate executive function, and the scoring criteria are complicated	Single‐domain	1
WMS	Roughly 120	Grades were determined by the performance on memory assessment scores	single‐domain	1

*Note*: “/” denotes an indefinite duration.

Abbreviations: AVLT, Auditory Vocabulary Learning Test; BNT, Boston Naming Test; CDR, Clinical Dementia Rating Scale; CDT, Clock Drawing Test; CERAD, Consortium to Establish a Registry for Alzheimer's Disease: Neuropsychological Assessment Battery; CVLT, California Verbal Learning Test; DST, Digit Span Test; HVLT, Hopkins Verbal Learning Test; LM, Logical Memory Test; MMSE, Mini‐Mental State Examination; MoCA, Montreal Cognitive Assessment; ROCF, Rey–Osterrieth Complex Figure Test; SCWT, Stroop Color and Word Test; SDMT, Symbol Digit Modalities Test; TMT, Trail Making Test; VFT, Verbal Fluency Test; WAIS, Wechsler Adult Intelligence Scale; WCST, Wisconsin Card Sorting Test; WMS, Wechsler Memory Scale.

### 3.4 Analysis of Subject Headings on Risk Factors for Cognitive Impairment in LLD

Utilizing the thematic analysis method, this study sorted out a total of five themes, namely (1) sociodemographic factors; (2) psychological factors; (3) physiological factors; (4) genetic factors; and (5) other factors. The frequency of inclusion for each variable is presented in detail in Table 3.

**TABLE 3:** The number of times each variable was included.

Subject headings for risk factors	Number of inclusions
Sociodemographic factors	
Age	7
Gender	3
Educational attainment	7
Financial capacity	1
Residence	2
Smoking	1
Psychological factors	
Vulnerability	1
Neuroticism and stress	2
Neuropsychiatric symptoms	3
Lower interpersonal support	1
Lower baseline global neuropsychological score	1
Physiological factors	
Underlying physical illness	5
Clinical manifestations of LLD	1
Microvascular dysfunction	1
Severity of baseline cognition and depression symptoms	3
Disease progression trajectory	2
Lack of regular physical activity	1
Genetic factors	
rs11666579 in SLC27A1	1
Methionine synthase (MTR) 2756AA polymorphism	1
Other factors	
Vitamin B_12_ deficiency	1
Elevated levels of NGAL	1
Cortisol level	1
BDNF	1
NRG‐1	1
SNRIs, TCAs, and benzodiazepines	1

Abbreviations: BDNF, brain‐derived neurotrophic factor; LLD, late‐life depression; NGAL, neutrophil gelatinase‐associated lipocalin; NRG‐1, neuregulin‐1; SNRIs, serotonin–norepinephrine reuptake inhibitors; TCAs, tricyclic antidepressants.

#### 3.4.1 Sociodemographic Factors

The risk factors for cognitive function decline in patients with LLD include increasing age (Funes et al. 2018; Gallagher et al. 2018; Li et al. 2018; Riddle et al. 2015; Shao et al. 2017; Steffens et al. 2020; Zhong et al. 2018), female gender (Funes et al. 2018; Gallagher et al. 2018; Li et al. 2018), education level (Funes et al. 2018; Gallagher et al. 2018; Li et al. 2018; Rej et al. 2015; Riddle et al. 2015; Steffens et al. 2020; Yu 2013), financial capacity (Morin et al. 2019), residing in rural or suburban areas (Li et al. 2018), living alone (Shao et al. 2017), and smoking habits (Shao et al. 2017). Furthermore, the role of financial capacity and management ability in the development of cognitive impairment among LLD patients has also been emphasized, particularly regarding executive function decline (Morin et al. 2019). In addition, smoking has also been identified as a significant risk factor for cognitive impairment in individuals with LLD (Shao et al. 2017).

Multiple studies have consistently demonstrated a robust association between cognitive impairment symptoms and lower education attainment, particularly illiteracy or elementary education, in older adults compared to individuals with higher levels of education such as junior high school or college (Funes et al. 2018; Gallagher et al. 2018; Li et al. 2018; Riddle et al. 2015; Steffens et al. 2020; Yu 2013). However, it was also reported no significant relationship between educational level and cognitive function (Rej et al. 2015).

#### 3.4.2 Psychological Factors

Psychological factors such as vulnerability (Manning, Chan, and Steffens 2017), neuroticism and stress (Manning, Chan, and Steffens 2017; Steffens et al. 2023), neuropsychiatric symptoms (Korten et al. 2014; Rej et al. 2015; Zhang et al. 2021), lower interpersonal support (Rej et al. 2015), and lower baseline global neuropsychological score (Rej et al. 2015) are associated with cognitive decline in patients with LLD. Specifically, neuroticism, including vulnerability (Manning, Chan, and Steffens 2017) or the interaction between stress and neuroticism (Manning, Chan, and Steffens 2017; Steffens et al. 2023), has a detrimental impact on global cognition. Moreover, neuropsychiatric symptoms, such as behavioral symptoms, mediate the negative effect on cognitive function in LLD patients while emotional apathy contributes to executive dysfunction in this population (Korten et al. 2014; Rej et al. 2015; Zhang et al. 2021). In addition, poor interpersonal support and lower baseline global psychological scores are positively correlated with a longer time of conversion to mild cognitive impairment (MCI)/dementia individuals with LLD (Rej et al. 2015).

#### 3.4.3 Physiological Factors

Various factors, including underlying physical illness (Li et al. 2018; Rej et al. 2015; Zhong et al. 2019), clinical manifestations of LLD (Wu et al. 2022), microvascular dysfunction (Zhou et al. 2021), severity of baseline cognition and depression symptoms (Korten et al. 2014; Shao et al. 2017; Yu 2013), hearing impairment (Gallagher et al. 2018), olfactory dysfunction (Zhang et al. 2022), disease progression trajectory (Gallagher et al. 2018; Korten et al. 2014), and lack of regular physical activity in daily life routines (Riddle et al. 2015) may exert an influence on cognitive function among individuals with LLD. The Cumulative Illness Rating Scale–Geriatrics (CIRS‐G) exhibited a positive correlation with the duration of conversion to MCI/dementia in patients with LLD (Rej et al. 2015). Hypertension, coronary heart disease, diabetes, and other cardiovascular diseases along with their associated risk factors significantly elevate the susceptibility to cognitive impairment in individuals diagnosed with LLD (Zhong et al. 2019). LLD patients presenting cardiovascular diseases or related risk factors demonstrated a 6.8‐fold higher likelihood of experiencing global cognitive decline compared to those without any such comorbidities during a 1‐year follow‐up period (Zhong et al. 2019). The clinical manifestations of LLD, such as somatic symptoms, suicidal tendencies, retardation of thinking, and reduced energy levels, were found to be negatively correlated with cognitive impairment (Wu et al. 2022). Notably, microvascular dysfunction such as enlarged perivascular space in centrum semiovale, may serve as an independent risk factor for cognitive impairment in LLD patients (Zhou et al. 2021). Some studies have demonstrated that prolonged or recurrent depressive episodes contribute to more severe cognitive decline (Shao et al. 2017). However, Korten et al. (2014) proposed that individuals experiencing their first‐episode of depression exhibit poorer episodic memory ability compared to those with recurrent episodes.

#### 3.4.4 Genetic Factors

A genome‐wide association study analysis revealed a significant association between rs11666579 in SLC27A1 and cognitive impairment in patients with LLD (Steffens et al. 2020). In addition, the methionine synthase (MTR) 2756AA polymorphism was identified as a predictor for a more pronounced decline in overall cognitive performance among LLD patients (Yang et al. 2017).

#### 3.4.5 Other Factors

Vitamin B_12_ deficiency (Gallagher et al. 2018) and elevated levels of neutrophil gelatinase‐associated lipocalin (NGAL) (Naudé et al. 2014) have been associated with an increased risk of cognitive impairment across specific domains. Cortisol level (Zhong et al. 2018), brain‐derived neurotrophic factor (BDNF), and neuregulin‐1 (NRG‐1) (Huang et al. 2021) may serve as independent risk factors for global cognitive decline in patients with LLD. The administration of serotonin–norepinephrine reuptake inhibitors (SNRIs), tricyclic antidepressants (TCAs), and benzodiazepines has been found to have a detrimental impact on cognitive performance (Korten et al. 2014).

## 4 Discussion

Cognitive dysfunction is a fundamental symptom of LLD and also serves as a significant risk factor for its recurrence, thereby profoundly impacting the quality of life of the elderly. The influencing factors are multifaceted and intricate, lacking systematic organization. Consequently, this employs evidence‐based methods to synthesize previous research and proposes that sociodemographic, physiological, psychological, genetic, and other related factors primarily contribute to cognitive dysfunction in LLD. Although cognitive function assessment tools specifically designed for individuals aged 18–65 years with major depressive disorder exist, there remains a dearth of reliability and validity tests for LLD.

### 4.1 The Current Dearth of Specific and Standardized Cognitive Function Assessment Tools for LLD Is a Notable Limitation in the Field

In recent years, there has been an increasing global focus on the mental health of older adults, leading to various national‐level initiatives. Simultaneously, significant attention has been directed towards cognitive impairment associated with mental health disorders. Despite the availability of numerous assessment tools for evaluating cognitive function in the elderly population, there remains a lack of specific screening instruments for LLD (Bakkour et al. 2014).

The MMSE, known for its user‐friendly nature and time efficiency, has emerged as the most commonly utilized assessment tool in clinical research, corroborating the findings of this scoping review. However, primarily designed for evaluating overall cognitive function, it is typically limited to providing a global score (Arevalo‐Rodriguez et al. 2021). Conversely, domain‐specific screening approaches can offer direct insights into a patient's cognitive abilities across various dimensions such as functionality and psychiatric aspects. Nevertheless, their implementation requires substantial time investment and professional healthcare assistance (Cullen et al. 2007), potentially resulting in higher dropout rates among elderly subjects in community settings. Furthermore, the lack of consensus on specific assessment tools for each cognitive dimension among individuals with LLD significantly impedes the effectiveness of large‐scale screening studies within communities.

McIntyre et al. (2017) proposed a novel online tool for assessing cognitive function in patients with depression. Subsequently, the Chinese version of this tool was translated and its reliability and validity were evaluated among depressed patients aged 18–65 years (Hou et al. 2020). However, further research is warranted to explore the applicability of this tool in geriatric depression.

### 4.2 To Elucidate the Detrimental Factors Contributing to Cognitive Impairment in Individuals With LLD

#### 4.2.1 Sociodemographic Factors

The findings from studies investigating the impact of sociodemographic factors on cognitive function in patients with LLD are generally consistent, except for the relationship between education level and cognitive function, which remains controversial. Several studies have demonstrated a significant association between lower education levels and cognitive impairment in patients with LLD (Funes et al. 2018; Gallagher et al. 2018; Li et al. 2018; Riddle et al. 2015; Steffens et al. 2020; Yu 2013). However, the study of Rej et al. (2015) found no independent association between education level and cognitive impairment. This discrepancy may be attributed to the fact that their study focused on the onset of MCI or dementia rather than utilizing a comprehensive neuropsychological assessment, potentially introducing bias with limited accuracy and sensitivity. Furthermore, previous research has suggested that educational attainment serves as an indicator of cognitive reserve and exerts a protective effect against Alzheimer's disease‐related decline but does not appear to directly influence overall cognitive function (Bhalla et al. 2005). Therefore, future investigations could consider selecting remitted geriatric depression as a focal point to mitigate potential confounding effects such as mood disorders.

#### 4.2.2 Psychological Factors

Previous studies have demonstrated that neuroticism and an escalation in stressful life events are associated with subsequent cognitive decline among older adults without depression (Dickinson et al. 2011; Luchetti et al. 2016). Furthermore, a significant correlation has been found between global cognitive decline in patients with LLD and vulnerability to stress (VS), as well as the neuroticism traits assessed by the NEO Personality Inventory–Revised (NEO PI‐R) (Manning, Chan, and Steffens 2017). In addition, there is evidence of an interactive effect between stressful life events and neuroticism on delayed memory decline in patients with LLD (Manning, Chan, and Steffens 2017). Neuropsychiatric symptoms, including anxiety, hallucinations, and irritability as behavioral symptoms, along with emotional factors such as anxiety, apathy, and sleep disorders, can exacerbate cognitive impairment in patients with LLD. These symptoms may also contribute to residual cognitive dysfunction during the recovery period (Funes et al. 2018; Korten et al. 2014; Yu 2013; Zhang et al. 2021). Among them, emotional symptoms may exert a greater influence on cognitive impairment during acute episodes. Conversely, behavioral symptoms aggravate cognitive impairment in the recovery state and potentially lead to persistent cognitive dysfunction (Zhang et al. 2021). The potential explanation lies in the stability of emotional symptoms during recovery periods in LLD, which allows persistent behavioral symptoms to assume a more prominent role. Furthermore, baseline scores of behavioral symptoms are predictive of cognitive decline (Zhang et al. 2021), highlighting the necessity for increased attention to psychological factors in clinical practice and providing valuable insights for the early development of precise treatment and rehabilitation strategies targeting cognitive function. In addition, given emerging evidence suggesting that sleep difficulties are an independent risk factor for cognitive impairment in LLD rather than solely a manifestation of depression, particular emphasis should be placed on addressing sleeping issues (Yu 2013).

#### 4.2.3 Physiological Factors

A higher burden of medical illness (Rej et al. 2015), poor physical health status (Li et al. 2018), and low levels of daily physical activity (Gallagher et al. 2018) can impact the cognitive function of LLD patients. Previous studies have demonstrated a direct association between cardiovascular disease and the development of Alzheimer's disease (Pasqualetti, Thayanandan, and Edison 2022), as well as vascular dementia (Wolters and Ikram 2019). Moreover, existing evidence suggests an increased risk of cognitive decline in individuals with LLD (Zhong et al. 2019). Therefore, it is crucial to develop safe and effective intervention methods for LLD patients based on high‐quality expert consensus and guidelines while continuously uncovering the underlying pathophysiological mechanism. In addition, the clinical manifestations of LLD including somatization symptoms, suicidal tendencies, retardation of thinking, and reduced energy levels were found to be negatively associated with cognitive impairment (Wu et al. 2022). The association between active depression within the past 2 years and an increased risk of cognitive decline has been established, although inconsistent findings exist regarding the impact of depression courses on cognitive function (Gallagher et al. 2018). Some studies suggest that prolonged or recurrent episodes are associated with more severe cognitive impairment compared to first episodes, while others indicate worse episodic memory in individuals experiencing their first episode possibly due to higher levels of worrying and motivational problems contributing to poor cognitive performance (Korten et al. 2014). However, discrepancies may also arise from variations in assessment tools and diagnostic indicators.

#### 4.2.4 Genetic Factors

Genetic factors may impact cognitive function in patients with LLD. The apolipoprotein E (APOE) (Lefterov et al. 2023) allele and MTHFR genotype (Beyer et al. 2003) have been identified as risk factors for Alzheimer's disease, while the MTR2756AA polymorphism may increase the risk of cognitive impairment in LLD patients (Yang et al. 2017). However, despite its significant association with Alzheimer's disease (Davies et al. 2014), the APOE allele does not appear to be associated with cognitive impairment in LLD patients (Yang et al. 2017). In contrast to candidate gene validation methods, genome‐wide association studies (GWAS) have the potential to identify novel genes and pathways related to cognitive function. Studies have demonstrated significant associations between gene polymorphisms such as rs11666579 in SLC27A1, rs73240021 in GRXCR1, and cognitive impairment in LLD (Steffens et al. 2020). Nevertheless, due to ethical considerations and other limitations, research on genetic factors affecting cognitive impairment in LLD remains insufficient. Therefore, further investigations and experiments are warranted to validate these findings.

#### 4.2.5 Other Factors

Elevated homocysteine (Hcy), increased NGAL concentration, elevated cortisol level, BDNF, and NRG‐1 have been identified as independent risk factors for cognitive impairment in LLD (Huang et al. 2021; Naudé et al. 2014; Zhong et al. 2018; Zhou et al. 2020). Assessing the corresponding blood indicators in patients can effectively predict the severity of cognitive decline. Further systematic investigation and understanding of mechanisms can provide cost‐effective and easily obtainable objective biomarkers for clinical practice. Hcy serves as a blood indicator reflecting vascular endothelial dysfunction, with increased levels associated with an elevated risk of cognitive decline. In addition, it may mediate the relationship between vitamin B_12_ and cognitive impairment (Gallagher et al. 2018). Furthermore, certain psychotropic medications such as TCAs, SNRIs, and benzodiazepines may be associated with poorer cognitive performance (Korten et al. 2014). Therefore, close monitoring is necessary to identify potential negative effects on cognition when prescribing psychiatric medications.

## 5 Limitations

Several limitations exist in this study: (i) the analysis of influencing factors on cognitive function did not differentiate between different courses of depression in LLD patients due to a scarcity of articles; (ii) the inclusion criteria were restricted to Chinese/English language publications, potentially affecting the comprehensiveness of the conclusions; (iii) subjectivity might have influenced the literature screening and theme division for identifying influencing factors. These limitations can be attributed to existing studies primarily focusing on acute‐onset depression, with relatively few studies on recovery episodes of LLD that pose challenges for classification.

## 6 Conclusion

Population aging, as a global phenomenon, has had a profound impact on both developing and developed countries. Consequently, the prevailing mental health issues among the elderly, such as depression, have garnered increasing attention. By employing evidence‐based practice methods through the approach of the scoping review, this study reveals a dearth of specific and standardized cognitive function assessment tools for LLD patients, potentially introducing a bias in cross‐comparisons. Furthermore, there is inconsistency in the findings regarding certain risk factors that affect cognitive function. To address these inconclusive results or unknown mechanisms associated with these factors, further extensive studies are warranted to clarify. As for modifiable risk factors like lifestyle and mood, subsequent research can develop precise intervention programs to guide clinical practice.

## Author Contributions

**Ping Jiang**: formal analysis, writing–original draft. **Yunfeng Gao**: formal analysis, data curation, funding acquisition. **Lin Wang**: data curation. **Xiaojun Shao**: formal analysis. **Lei Zhang**: formal analysis, software. **Gang Zhu**: funding acquisition, writing–review and editing, resources. **Li Duan**: writing–review and editing, supervision, funding acquisition, project administration, methodology.

## Conflicts of Interest

The authors declare no conflicts of interest.

### Peer Review

The peer review history for this article is available at https://publons.com/publon/10.1002/brb3.70265.

## Data Availability

The data that support the findings of this study are available from the corresponding author upon reasonable request.

## Appendix A

### Search Strategy

#### Searches conducted November 2023

**Pubmed search**:

(((cognitive impairment [Title/Abstract]) OR (cognitive dysfunction [MeSH Terms])) OR (cognitive decline [Title/Abstract])) AND ((late‐life depression [Title/Abstract]) OR (geriatric depression [Title/Abstract]))

**Embase search**:

#1 cognitive impairment [Title/Abstract] OR cognitive dysfunction [Title/Abstract] OR cognitive decline [Title/Abstract]

#2 late‐life depression [Title/Abstract]) OR geriatric depression [Title/Abstract]

#3 depress*[Title] OR depressive disorder [Title]

#4 elderly [Title/Abstract]

#5 #3 AND #4

#6 #2 OR #5

#7 #1 AND #6

**Web of Science**:

#1 cognitive impairment [Topic] OR cognitive dysfunction [Topic] OR cognitive decline [Topic]

#2 late‐life depression [Topic]) OR geriatric depression [Topic]

#3 #1 AND #2

Filters applied: English language

**CINAHL**:

#1 cognitive impairment [Abstract] OR cognitive dysfunction [Abstract] OR cognitive decline [Abstract]

#2 late‐life depression [Abstract]) OR geriatric depression [Abstract]

#3 #1 AND #2

Filters applied: English language; Age (45–64 years, 65+ years)

**APA PsycInfo**:

#1 cognitive impairment [Abstract] OR cognitive dysfunction [Abstract] OR cognitive decline [Abstract]

#2 late‐life depression [Abstract]) OR geriatric depression [Abstract]

#3 #1 AND #2

Filters applied: English language;Age (40–64 years, 65+ years)

**Ovid**:

#1 cognitive impairment [Abstract] OR cognitive dysfunction [Abstract] OR cognitive decline [Abstract]

#2 late‐life depression [Abstract]) OR geriatric depression [Abstract]

#3 #1 AND #2

**CNKI**:

#1 cognitive impairment + cognitive dysfunction + cognitive decline

#2 late‐life depression + geriatric depression

#3 influencing factor

#4 #1 AND #2 AND #3

#### VIP

#1 cognitive impairment (Title/Abstract) OR cognitive dysfunction (Title/Abstract) OR cognitive decline (Title/Abstract)

#2 late‐life depression (Title/Abstract) OR geriatric depression (Title/Abstract)

#3 influencing factor (Title/Abstract)

#4 #1 AND #2 AND #3

#### Wanfang Database

(Abstract: (cognitive impairment) or Abstract: (cognitive dysfunction) or Abstract: (cognitive decline)) and Abstract: (late‐life depression)

#### SinoMed

#1 depressive disorder (Mesh)

#2 elderly (Mesh)

#3 #1 AND #2

#4 cognitive dysfunction (Mesh) OR cognitive decline (Common field) OR cognitive impairment (Common field)

#5 #3 AND #4
